# Pattern Recognition Approaches for Breast Cancer DCE-MRI Classification: A Systematic Review

**DOI:** 10.1007/s40846-016-0163-7

**Published:** 2016-08-31

**Authors:** Roberta Fusco, Mario Sansone, Salvatore Filice, Guglielmo Carone, Daniela Maria Amato, Carlo Sansone, Antonella Petrillo

**Affiliations:** 1 Department of Diagnostic Imaging, metabolic and radiant Therapy, National Cancer Institute of Naples “Pascale Foundation”, Via Mariano Semmola 80131, Naples, Italy; 2 Department of Electrical Engineering and Information Technologies, University ‘Federico II’, Via Claudio 80125, Naples, Italy

**Keywords:** Dynamic contrast-enhanced magnetic resonance imaging (DCE-MRI), Breast cancer, Patter recognition approach, Classification

## Abstract

We performed a systematic review of several pattern analysis approaches for classifying breast lesions using dynamic, morphological, and textural features in dynamic contrast-enhanced magnetic resonance imaging (DCE-MRI). Several machine learning approaches, namely artificial neural networks (ANN), support vector machines (SVM), linear discriminant analysis (LDA), tree-based classifiers (TC), and Bayesian classifiers (BC), and features used for classification are described. The findings of a systematic review of 26 studies are presented. The sensitivity and specificity are respectively 91 and 83 % for ANN, 85 and 82 % for SVM, 96 and 85 % for LDA, 92 and 87 % for TC, and 82 and 85 % for BC. The sensitivity and specificity are respectively 82 and 74 % for dynamic features, 93 and 60 % for morphological features, 88 and 81 % for textural features, 95 and 86 % for a combination of dynamic and morphological features, and 88 and 84 % for a combination of dynamic, morphological, and other features. LDA and TC have the best performance. A combination of dynamic and morphological features gives the best performance.

## Introduction

Breast cancer is the most common cancer among women in the Western world. It is the second leading cause of cancer death in women today (after lung cancer) and is estimated to cause 15 % of cancer deaths [1]. Therefore, screening for early diagnosis of breast cancer is of great interest.

The currently widespread screening method is RX mammography, which plays an important role in clinical practice [2, 3]. However, this method has some drawbacks: it uses ionizing radiation, it is not adequate for young women because of their high-density breasts, and detection of breast lesions is difficult because of the lack of functional information. Breast ultrasound (US) is able to detect additional cancers in women with dense breasts and negative mammography and is helpful for the characterization of mammographically detected abnormalities, evaluation of tumor size and nodal status, and guiding needle biopsy [4]. However, it is of limited value in detecting additional ipsi- or contra-lateral malignant lesions.

Magnetic resonance imaging (MRI) and in particular the emerging methodology of dynamic contrast-enhanced (DCE)-MRI has demonstrated great potential in the screening of high-risk women, staging newly diagnosed breast cancer patients, and assessing therapy effects thanks to its minimal invasiveness and ability to visualize dynamic (functional) information not available with conventional imaging. Therefore MRI, and in particular DCE-MRI, is gaining popularity as an important complementary diagnostic tool for the early detection of breast cancer [2–5].

MRI is currently used as a complement to conventional X-ray mammography in the diagnosis of breast lesions [2]. It has been shown that 17–34 % of cancer foci visible on breast MRI are not detected by mammography. Because of the higher cost and increased time required to read an MRI data set (~400 images per patient), MRI will probably never be a complete replacement for mammography, but it is certainly an excellent screening tool for high-risk patients. Reducing the workload required to read an MRI data set would make it a more practical clinical screening tool. Therefore, the development of methods using low-cost hardware for lesion detection and classification is of great interest. X-ray mammography remains the gold standard for breast cancer screening and offers high two-dimensional (2D) resolution, which is advantageous for detecting small variations in tissue composition, such as micro-calcifications [3].

However, due to the constraints of imaging a three-dimensional (3D) structure in a single plane, breast US or DCE-MRI is often used as a secondary imaging technique when a suspicious lesion is found using mammography [3, 5]. DCE-MRI is also very good at imaging dense breasts, but its major advantages over mammography and US are the ability to (a) image the entire breast as thin slices that comprise the entire breast volume and (b) measure variations in contrast uptake that provide information about the vascularity of the breast tissue [6].

On account of breast DCE-MRI’s high 3D resolution and its ability to acquire kinetic contrast information, its lesion detection sensitivity is close to 100 % [7], much higher than that of either mammography or US [1]. However, the specificity of breast DCE-MRI is low, with reported rates of between 30 and 70 % [7, 8].

In addition to the problem of low specificity, another shortcoming of breast MRI is that only experienced radiologists are able to accurately distinguish benign from malignant tumors [1, 9]. This often leads to high rates of inter-observer variability [9]. Therefore, one of the challenges in facilitating increased acceptance of breast DCE-MRI as a screening modality is reducing false positive detection errors, thereby boosting detection specificity. Additionally, the inter-observer variability for breast DCE-MRI must be minimized.

For these reasons, several authors have proposed using various features in DCE-MRI images to decide whether a given tumor is benign or malignant. For example, radiologists differentiate tumors based on features that describe the biological activity of the tumor using dynamic parameters (vascularization, permeability, flux) [10–22], tumor size, tumor boundary shape (morphological characteristics), or tumor heterogeneity (textural features) [23–49].

Computer-aided diagnosis (CAD) systems, using pattern analysis approaches, have the potential to assist radiologists in the detection and classification of breast cancer. A key component of the development of such CAD systems is the selection of an appropriate classification function responsible for separating malignant and benign lesions.

In the last two decades, many studies have addressed the problem of tumor lesion classification based on DCE-MRI data analysis. It is has been recognised that this problem can be addressed in a pattern recognition framework with the use of opportune features and classifiers.

Despite large effort, there is still no agreement on the features most suitable for this task. Many kind of features have been used. Dynamic features take into account the time course of the contrast agent within the lesion, but they can fail to describe other features of the lesion, such as tumor heterogeneity. Textural features have thus been introduced. Compartmental modeling can add useful information concerning vascular permeability. Furthermore, morphological features have been traditionally used in tumor classification and they can be added to other dynamic features, with the additional advantage that tumor morphology can be delineated more precisely using the dynamic information of DCE-MRI. Spatiotemporal features have been suggested to combine spatial and dynamic information.

Similarly, it is not clear what kind of classifiers can give the best performance. Several classifiers have been used. It is generally recognised that tree-based classifiers are more easily accepted by humans because they can require to simple threshold-based rules; however, they can suffer from over-fitting. Linear classifiers are also easy to design and understand, but linear combinations of features do not always have simple interpretations. More sophisticated classifiers such as artificial neural networks (ANN) and support vector machine (SVM) are strongly non-linear and thus classification hyperplanes are difficult to interpret. Moreover, not all classifiers work well with all types of features; therefore, various combinations of classifier-features have been attempted.

This study surveys the literature of the last two decades that focuses on features and classifiers used for the classification of tumor lesions detected from DCE-MRI data. We performed a systematic review of several machine learning algorithms proposed in the literature for classifying breast lesions using dynamic [10–22], morphological, and textural features in DCE-MRI [23–49]. This study considers the following machine learning approaches: ANN, SVM, linear discriminant analysis (LDA), tree-based classifiers (TC), and Bayesian classifiers (BC). This systematic review is conducted using a meta-analysis. As such, our objective is not to present a short summary of all studies, but instead to focus on aspects common to all studies and present a statistical analysis of the performance of the algorithms in the literature.

## Review of Methodology

### Search Criteria

Several electronic databases were searched, namely PubMed (US National Library of Medicine, http://www.ncbi.nlm.nih.gov/pubmed), Scopus (Elsevier, http://www.scopus.com/), Web of Science (Thomson Reuters, http://apps.webofknowledge.com/), and Google Scholar (https://scholar.google.it/). The following search criteria were used: “breast cancer” and “breast lesions” for the clinical domain and “DCE-MRI”, “Dynamic Contrast Enhanced-MRI”, and “Dynamic Contrast Enhanced-Magnetic Resonance Imaging” for the diagnostic test. To make sure that no study was missed, a free-text search was also performed. The search covered the years from 1995 through 2014. Furthermore, all reference lists of the obtained papers were scrutinized for studies not indexed in the electronic databases.

If not otherwise stated, all the studies reviewed herein fulfill the following criteria: (1) thorough clinical characterization of the patients with DCE-MRI (studies using other diagnostic techniques were excluded); (2) specification of applied classifiers; (3) accuracy of classifier reported in terms of sensitivity and specificity; and (4) used one of the following classifiers: ANN, SVM, LDA, TC, or BC.

**Fig. 1:**
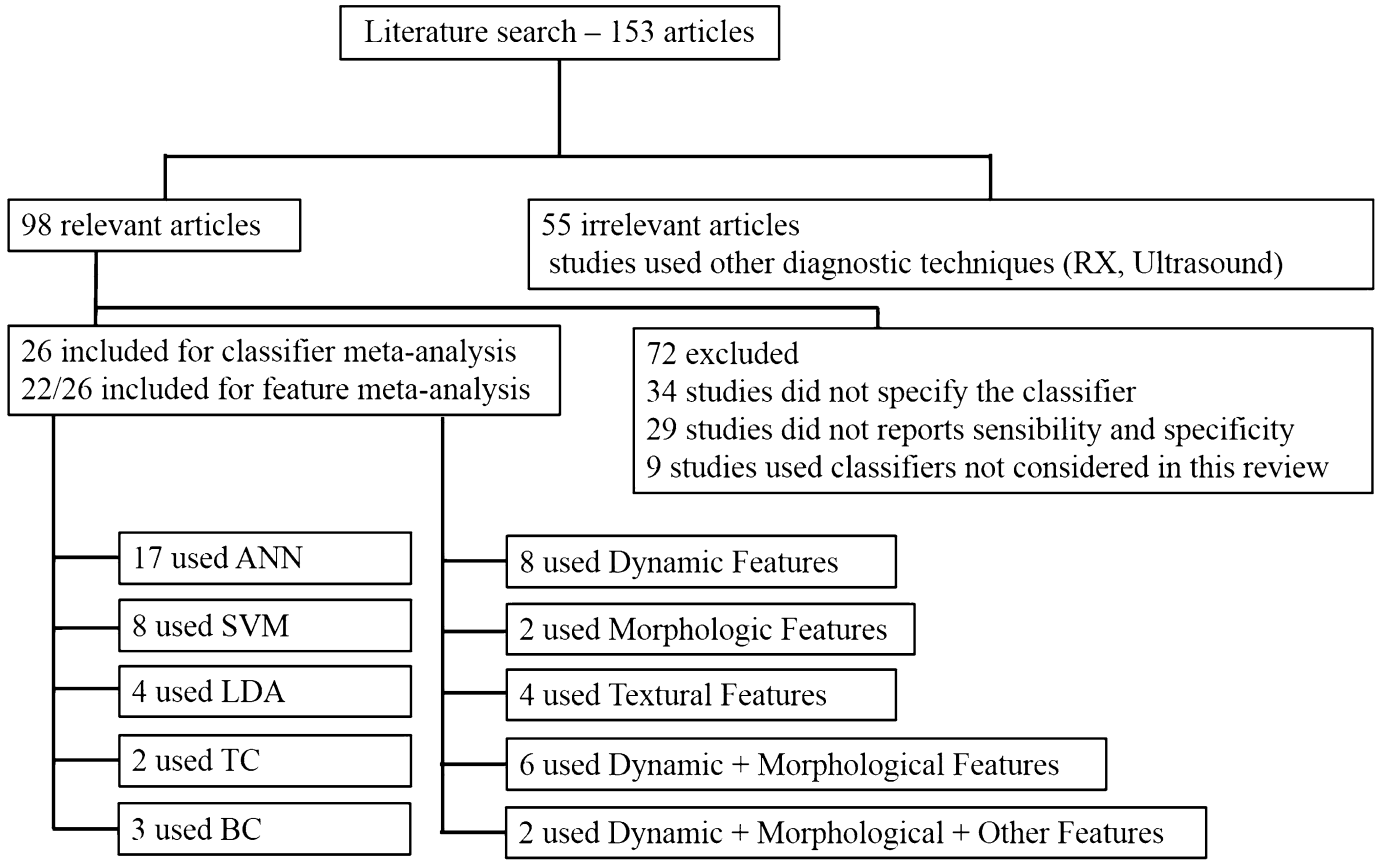
Included and excluded studies in systematic review

In the present review, all relevant studies were scrutinized, but only studies that satisfied the inclusion criteria are included in the review (Fig. 1). Furthermore, this analysis was carried out only for studies of subjects with breast lesions. Information extracted from each study included the title, authors, year of publication, sample size, age of subjects, reference standard, and numbers of true positives (TP), false positives (FP), true negatives (TN), and false negatives (FN).

### Pattern Recognition Approach

As many textbooks are available on this subject [34–39], only a brief and informal description of the main concepts is given.

Using the pattern recognition approach, the subjects are divided into different classes, each one characterized by different features (dynamic, morphological, textural, clinical, spatiotemporal, pharmacokinetic). The classes were formed in such a way that individuals belonging to a given class were characterized by similar values, occupying a region in the multidimensional feature space, well separated from the other classes.

Pattern recognition methods are based on three main phases: feature extraction/selection, training, and classification. While the training and classification tasks can be considered a well-defined area, the extraction/selection of the most appropriate features for a specific field of research must be delineated specifically.

In the first phase, feature extraction means that existing features are combined to produce new ones. Several methods can be used to achieve this task. The main problem is that during the transformation (linear or non-linear feature combination), the physiological meaning of the original features may be lost.

In feature selection, only some of the features are chosen to eliminate redundant features, thus improving system efficiency. Two types of approach can be used for feature selection, namely wrapped and filter methods. A wrapper method uses a specific classifier to evaluate the features. This leads to high performance, since the selected features are the most appropriate for the chosen classifier. The filter method does not take into account the classifier. It is thus less computationally expensive when the number of features is very large.

After feature extraction/selection, testing is conducted, in which the classifier is designed using a training data set with the characteristics of the population under investigation. The classifier first has to be proven using a testing data set, which is different from the training data set. The performance of the classifier and its sensitivity to the training and testing data sets can be analyzed using two methods: the leave-one-out method and the 10-fold method.

The leave-one-out method removes elements from the data set, one at a time. Then, the classifier is designed based on the remaining elements and is tested using the removed ones. This method can only be used on a small database. For the 10-fold method, the data set is divided into 10 subsets, and then a procedure similar to that of the leave-one-out method is applied.

For the final validation, a validation data set, which is different from both the training and testing data sets, is typically aplied. The final phase is classification. Various machine learning algorithms can be used, such as ANN, SVM, LDA, TC, and BC.

A widely used strategy is to consider different classifiers at the same time. Each classifier receives the same set or subset of features as input and the final decision on the class is taken using an adequate scheme.

### Classifiers

In this survey, we focus on the most commonly used classifiers, namely ANN, SVM, LDA, TC, and BC. A brief informal description of each classifier is given. The theoretical details of these classifiers can be found elsewhere [26–29].

#### Artificial Neural Networks

ANN is a set of mathematical models that mimic the behavior of neurons in the human brain, connected to each other through synapses. A neural network is a collection of elements (neurons) that are individually able to perform a fairly simple task and are interconnected with each other through unidirectional channels in order to perform more complex behavior. The output signal of the network is calculated on the basis of an input signal (feature vector) and the local memory of each neuron. An intermediate hidden layer of neurons is applied in classification problems that cannot be solved by a single-layer network. An input-hidden-output structure is called a multilayer perceptron (a single-layer network is called a perceptron). The set of inputs and the contents of the local memory are considered the inputs of a suitable transfer function that calculates the output, which will be propagated to other neurons, and so on, until it reaches the final output of the network [34–39]. This architecture is capable of drawing a hyperplane in the feature space that separates the classes. Typically, it achieves this task using an algorithm for updating weights called back propagation [34–39].

#### Support Vector Machines

SVM is a binary classifier that separates data using a hyperplane, determined based on selected points from the training set. While the traditional methods for classification are based on the minimization of empirical risk, or the optimization of performance on the training set, SVM minimizes the structural risk, i.e., the probability that new samples are classified correctly for a fixed probability distribution of the data.

Given a set of linearly separable data, there are various hyperplane separators that discriminate the data correctly. SVM identifies the hyperplane that, in addition to being correct for the training set, is also able to maximize the margin, defined as the sum of the distances between the hyperplane and the nearest points on both sides of it. Such points are called support vectors and are the only points of the training set used for determining the optimal hyperplane [34–36].

#### Linear Discriminant Analysis

LDA is a method of classification whose basic idea is to build decisional contours to separate the objects of the classes using the optimization of the error criterion. The method is based on the fact that the distributions of data with a large variance between two classes and those with a smaller variance within each class are easy to separate [34–37].

#### Tree-Based Classifiers

A TC is based on the idea of dividing a complex decision into a union of many easier ones, so that at the end, the solution obtained reflects the desired one. This kind of classifier has the advantage of being very fast. A TC simplifies complex calculations and deletes unnecessary ones, and is also very intuitive and easily understandable [34–36].

#### Bayesian Classifiers

A Bayesian network is a probabilistic graphical model of knowledge in an uncertain domain that can be used to build a BC, which estimates statistical data from the training set to calculate the posterior probability. The main advantages of BC are a simple association between the knowledge of the model and that of the data, and ability to model reality in conditions of uncertainty [34–36].

### Features for Breast Classification

#### Dynamic Features

Dynamic features (DYN) describe the temporal dynamics of the signal through measures obtained directly from the time-intensity curve. They are therefore model-free, since they are not calculated according to a model. The main dynamic features are area, maximum intensity ratio, relative enhancement, relative enhancement slope, basal signal, perfusion index, sum of intensities difference (SOD), wash-in, wash-out, and time to peak [41, 42, 49].

#### Pharmacokinetic Features

Pharmacokinetic features (PK) reflect some physiological parameters of tissues and are calculated on the basis of mathematical models according to a model-based strategy [45–48]. They include extracellular extravascular space (EES), plasma space, and transfer constants between the plasma space and the EES. Moreover, when more complex kinetic models were used, pharmacokinetic features also include permeability flux, extraction fraction, and capillary transit time [45–48].

#### Spatiotemporal Features

Spatiotemporal features (STEP) model the signals in a four-dimensional space to capture not only the temporal dynamics and the architectural characteristics, but also the spatial variations of the voxels. Spatial and temporal properties are combined to obtain these features [17, 33].

#### Morphological Features

Morphological features (MOR) describe the shape and structure of the region of interest obtained in detection. The main morphological features are area, circularity, compactness, complexity, perimeter, radial length, smoothness, roughness, sphericity, eccentricity, volume, rectangularity, solidity, speculation, convexity, curvature, and edge [25, 32, 40, 41].

#### Textural Features

Textural features (TEX) are based on the texture of the image, i.e., its geometric structure. There are many definitions of texture; in general terms, it can be seen as a function of local spatial variation in the intensity of the voxels. Therefore, textural features replace the original values of the voxels with measures that describe their statistical properties: mean, median, standard deviation, kurtosis, and skewness [43, 44].

#### Clinical Features

Clinical features (CLI) relate to the patient’s medical records and can provide additional information or instructions that may be useful for classification [31].

### Data Analysis

All data analysis were performed using the software RevMan (version 5.2) [50]. Forest plots were constructed to graphically present the sensitivity and specificity values, with corresponding 95 % confidence intervals (CIs), for the individual studies. A summary receiver operating characteristic (sROC) curve was constructed using the same software.

## Results

By using the search terms described earlier, we identified 153 studies from 1995 through 2014. Of these, 55 studies used diagnostic techniques other than DCE-MRI, 34 studies did not specify which classifiers were used, 29 did not have sufficient data (did not report sensitivity and specificity), and 9 studies used classifiers that were excluded from this review. 26 studies remained for inclusion in our meta-analysis, 20 were performed after 2000 (Fig. 1).

**Fig. 2:**
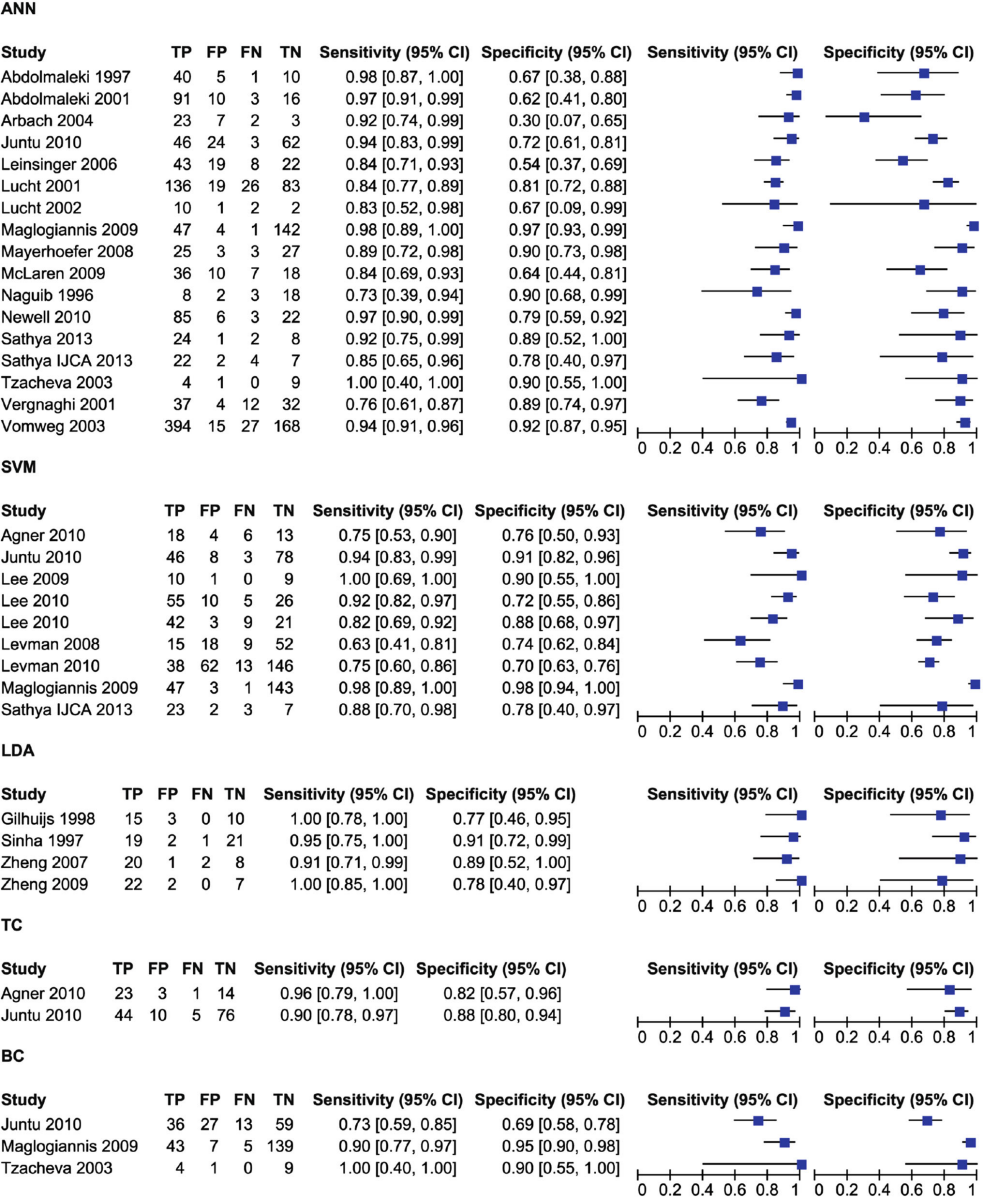
Forest plot of sensitivity and specificity, with corresponding 95 % CIs, of included studies, divided by classifiers

**Fig. 3:**
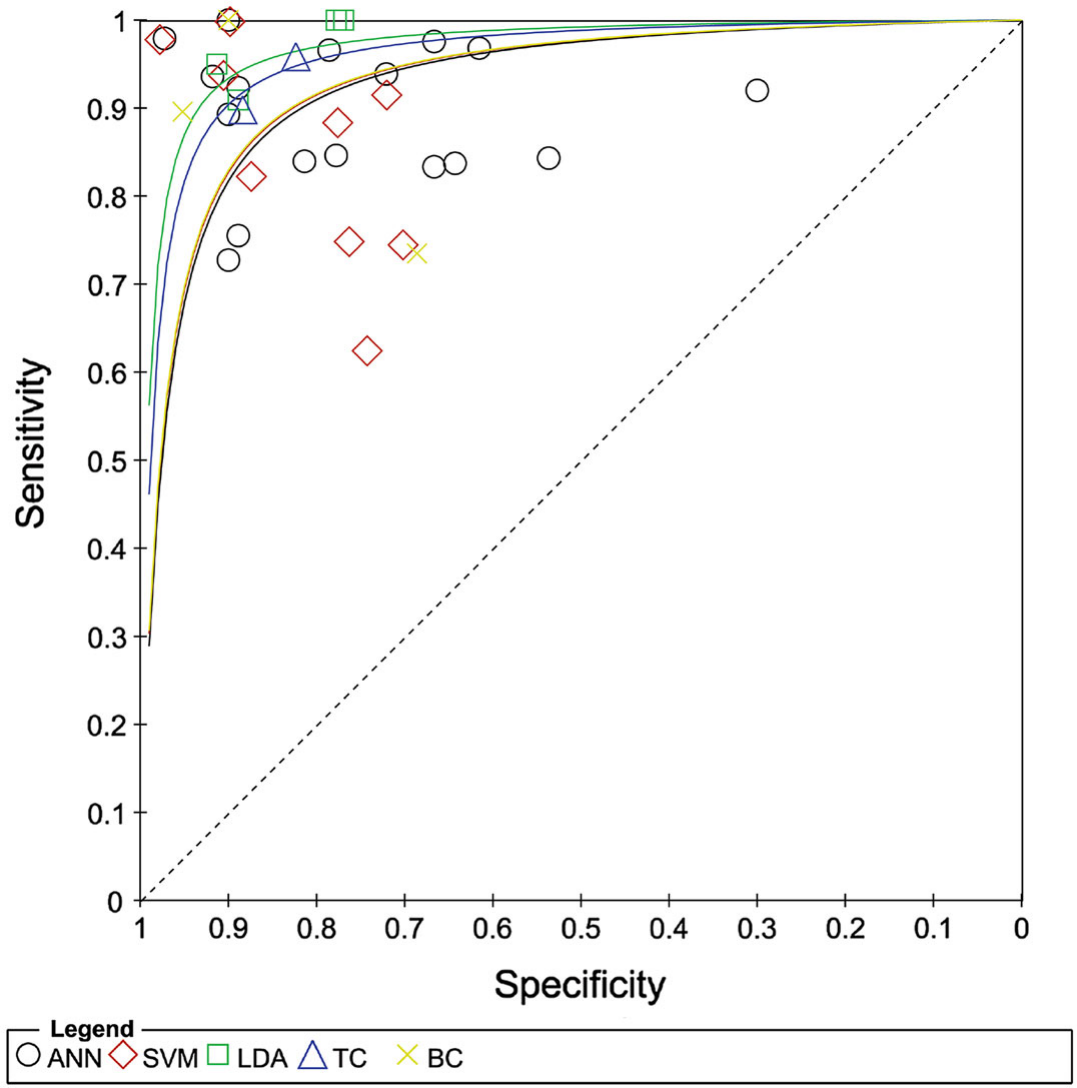
Sensitivity and specificity plotted in receiver operating characteristic space for individual studies; sROC curves are plotted from data points for each classifier

As shown in Table 1, the studies included in this review are divided by classifier as follows: 17 used ANN, 8 used SVM, 4 used LDA, 2 used TC, and 3 used BC. Some studies used multiple classifiers. The results of this first meta-analysis are shown in Figs. 2 and 3. Figure 2 shows the values of TP, FP, FN, TN, sensitivity, and specificity for each study, divided according to the applied classifier. Figure 3 shows the sROC curves for each classifier.

**Table 1:** Numbers of studies and patients per classifier

Classifier	Number of studies	Total number of patients
ANN	17	1960
SVM	8	949
LDA	4	133
TC	2	176
BC	3	343

**Fig. 4:**
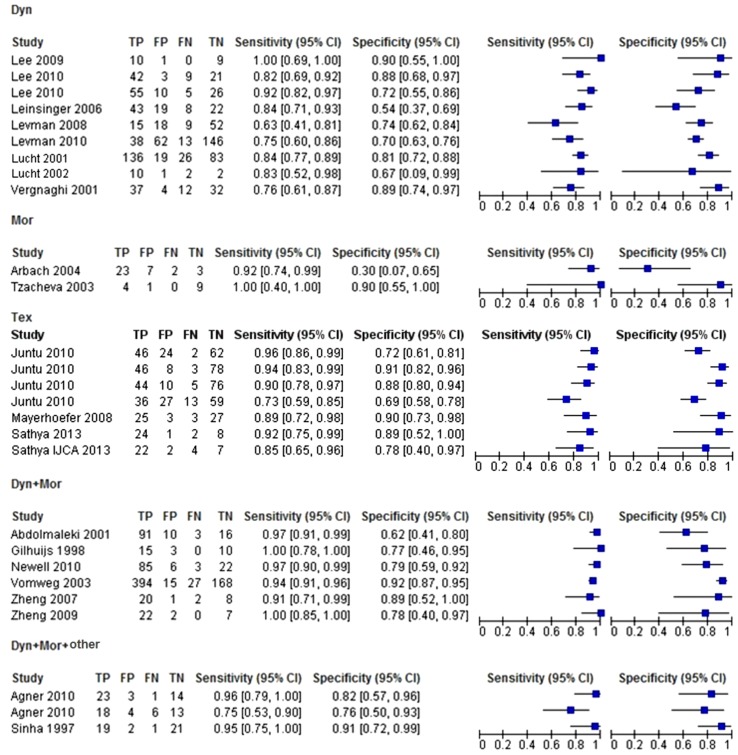
Forest plot of sensitivity and specificity, with corresponding 95 % CIs, of included studies, divided by features

**Fig. 5:**
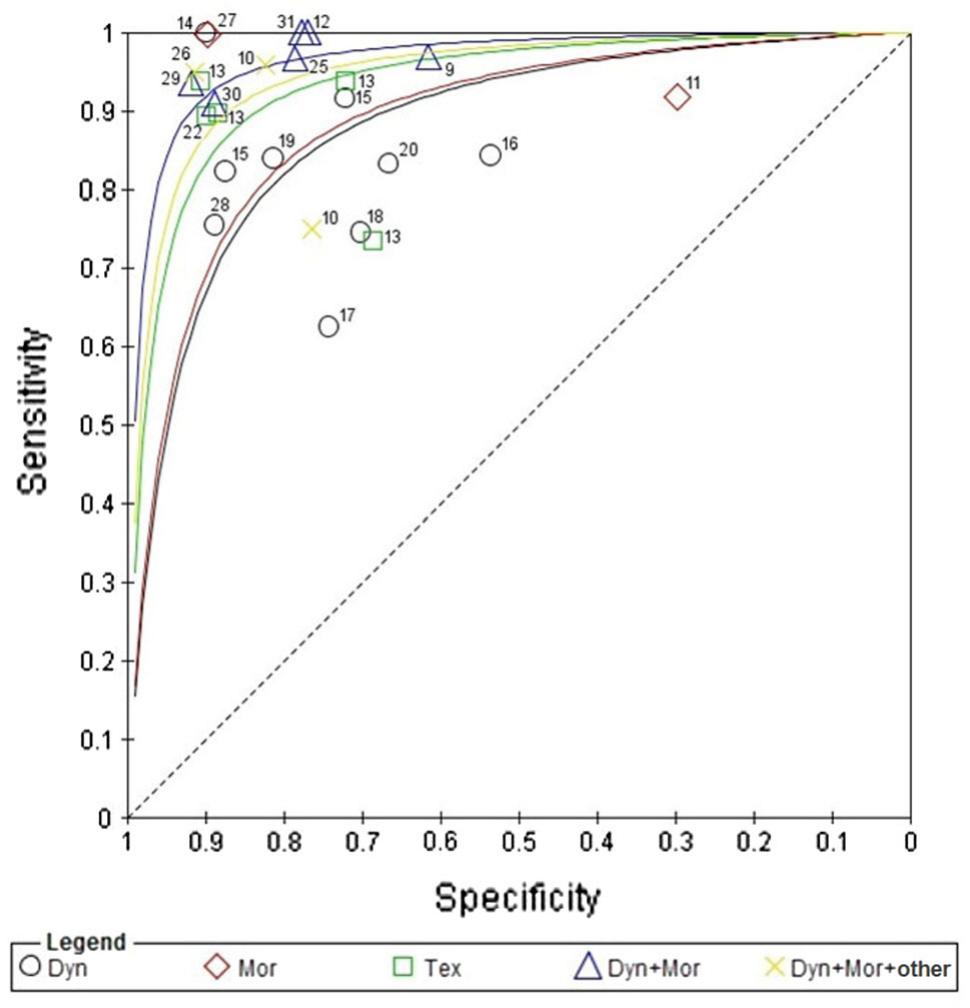
Sensitivity and specificity plotted in receiver operating characteristic space for individual studies; sROC curves are plotted from data points for each feature

The included studies also considered different features. In this second systematic meta-analysis, we considered only the studies that reported a detailed description of used features (22 of the 26 included studies). The most used features were dynamic features, followed closely by morphological features, and then textural features (Table 2). The results of this second meta-analysis are shown in Figs. 4 and 5, which respectively show the forest plot and the sROC curves.

**Table 2:** Numbers of studies and patients per feature

Feature	Number of studies	Total number of patients
DYN	8	1000
MOR	2	49
TEX	4	668
DYN + MOR	6	930
DYN + MOR + other	2	125

## Discussion

In the last two decades, many studies have tackled the problem of tumor lesion classification based on DCE-MRI data analysis. It is has been recognized that this problem can be addressed in a pattern recognition framework with the use of suitable features and classifiers. The present study performed a systematic review of several machine learning algorithms proposed in the literature to classify breast lesion using several features categories such as dynamic, morphological, and textural features in DCE-MRI. Our results indicate that the choice of features does not affect the selection of the classifier; in fact, many authors used different combinations of features and classifiers, without compromising the validity of their study. We can thus safely say that the choice of features and that of classifier must not necessarily be related.

Although machine learning algorithms heavily depend on the training data and extracted features and the reported sensitivities and specificities cannot be compared directly, we can draw some conclusions. Based on the collected data, the averagevalues of sensitivity and specificity were calculated for each classifier. The sensitivity and specificity were respectively 91 and 83 % for ANN, 85 and 82 % for SVM, 96 and 85 % for LDA, 92 and 87 % for TC, and 82 and 85 % for BC.

The average values are 90 % for sensitivity and 83 % for specificity. At first, it might seem that LDA and TC have the best performance (Fig. 2); however, from Table 1, these two classifiers have the smallest samples of patients. In fact, LDA was used in only 4 studies, with a total of 133 patients, and TC was used in only 2 studies, with for a total of 176 patients.

In Fig. 3, the sROC curves corresponding to the SVM, ANN, and BC classifiers are in red, black, and yellow, respectively. These three curves are almost superimposed. A few studies achieved very good performance in term of both sensitivity and specificity. Considering all the studies involving ANN, SVM, and BC, these classifiers appear to have similar performances. However, these performance seem less precise than those seen previously; this probably stems from the fact that the statistical samples used in these cases are larger, and thus more accurate estimations of performance were obtained.

We now discuss the results of our second meta-analysis. From Fig. 4, we calculated the average values of sensitivity and specificity for each type of feature. The sensitivity and specificity are respectively 82 and 74 % for dynamic features, 93 and 60 % for morphological features, 88 and 81 % for textural features, 95 and 86 % for a combination of dynamic and morphological features, and 88 and 84 % for a combination of dynamic, morphological, and other features.

In Fig. 5, the sROC curves show that dynamic and morphological features used alone have similar performances, but they are not precise. Better performance can be achieved using multiple features simultaneously. In Fig. 5, the least and most accurate curves are for studies that used only dynamic features and those that used a combination of dynamic and morphological features, respectively. Table 2 shows that 8 studies used only dynamic features, with a total of 1000 patients, and that 6 studies used a combination of dynamic and morphological features, with a total of 930 patients. The numbers of statistical samples in these cases are very similar and thus the performance improvement is associated with the use of more kinds of feature.

The contribution of literature surveys in general and of systematic reviews in particular, is to collect much information (in this case, types of classifiers, types of features, results on small and large populations, etc.) in a single place. This study adopted a systematic approach to summarize the performances (weighted on the basis of population size) of many studies, giving an overall indication of which methods can give good results. It must be underlined that even if a technique shows promising results for a small patient population, it must be verified with larger samples before it can become a standard protocol that clinicians can use in routine examinations.

## Conclusion

This study performed a systematic review of several pattern analysis approaches for classifying breast lesions using dynamic, morphological, and textural features in (DCE-MRI) images. Our results indicate that LDA and TC have the best performance and that the remaining classifiers analyzed in this review (ANN, SVM, and BC) have similar performances, but are less precise than LDA and TC. This probably stems from the fact that the numbers of statistical samples used for the latter classifiers are larger, allowing a more accurate analysis. Moreover, dynamic and morphological features achieve better performance when used simultaneously in a given classifier.

One of the main issues that emerged from this study is the lack of standardization of the breast MRI exam. The scanning protocols for breast MRI vary in terms of pulse sequence parameters, spatial and temporal resolutions, field of view, exam duration, contrast agent dose injected, type of infusion, image pre- and post-processing, etc. This number of variables makes it difficult to compare studies. Efforts at an international level should be directed toward the assessment of guidelines.

A second issue related to the previous one is the lack of a publically available database of breast MRI images for the assessment of pattern recognition algorithms for feature extraction and classification. Such a database will improve the evaluation of algorithms for image processing, feature extraction, and classification. Moreover, small sample sizes, which is an issue in a large number of the examined studies, could be addressed in this way.

A third issue is the variety of feature types used in previous studies. The majority of formulas used in previous studies try to extract information from the time course of the contrast agent and from the heterogeneity of the tumor. However, often, because of differences in MRI scanning protocols, the mathematical formulas of certain features cannot be directly used in different MRI settings. Moreover, blindly mixing features is not always a good approach because the mixtures cannot be properly understood by the clinician. An optimal set of features should be capable of effectively classifying tumors, be used in every MRI setting, and be as simple as possible to make sense for radiologists.

If the space of features is good, the project of the classifier would be simplified (e.g., linear or minimum distance). However, it is in general possible to obtain better performance by using more sophisticated classifiers (ANN, SVM, etc.) in combination with mixed features. The risk in this case is that an incomprehensible (to humans) set of features could emerge in combination with a strongly non-linear classifier. The adequacy of such a situation is questionable.
